# Supplementary material for: Are carbon emissions trading and green financial instruments synergistic? -Comprehensive quantitative research based on content analysis
Source: PLoS One. 2024 Mar 7;19(3):e0298601. doi: 10.1371/journal.pone.0298601 (PMC10919601; doi:10.1371/journal.pone.0298601)
Supplement: S1 Data — (ZIP) [file pone.0298601.s001.zip › Data/地级市Z_F工作报告.zip!/╣π╢1⁄2/├╖╓▌╩╨/└┤╫╘╣1⁄2╓┌║┼í╛┬φ┐╦╩2╛▌═°í┐_2013.docx]

各位代表：

　　我代表梅州市人民政府向大会作报告，请予审议。并请政协委员和其他列席人员提出意见。

　　2012年工作回顾

　　2012年，面对经济下行的较大压力，市政府坚持调结构、稳增长、惠民生、促和谐，较好地完成了市六届人大一次会议确定的目标任务。全市生产总值、公共财政预算收入、固定资产投资分别达745.98亿元、56.27亿元和230.14亿元，比上年分别增长10.7%、20.0%和16.4%，增速均超过全省平均水平。在广东省群众幸福感测评中，我市居粤东西北首位，并荣获“中国最具幸福感城市”称号。

　　一、经济发展稳中有进

　　经济总量和质量有新提升。加快烟草、电力、建材等传统产业改造升级，三次产业比例调整为21.3∶36.4∶42.3。深入开展 “暖企行动”，支持民营经济发展。全市工业增加值227.3亿元，比增14.7%。社会消费品零售总额403.5亿元，比增11.8%；进出口总额15亿美元，比增10.1%。我市银行存款突破千亿元，被评为全省首个“金融生态市”，被确定为“粤东北与港澳金融合作试验区”，农村金融改革创新工作经验在全省推广。

　　园区建设和招商引资有新成效。高新技术产业园区迁址和广州 （梅州）产业转移园扩园工作获省批准，园区开发面积拓展到11.76平方公里，一批项目建成投产。东莞石碣（兴宁）产业转移园、五华经济开发区等工业园区建设步伐加快。大力实施乡贤回乡投资兴业工程，引进了法国欧尚、香港毅德等世界500强企业和伊利集团、大百汇等一批优质项目。

　　文化旅游特色区建设有新进展。出台了创建广东梅州文化旅游特色区的决定。完成了雁洋核心区的规划，启动了一批重点项目的建设。成功举办首届客家文化艺术节，彰显了世界客都风采，初步打响了“休闲到梅州，享受慢生活”品牌。继梅县之后，大埔成功创建省旅游强县。全市旅游接待人数和旅游总收入分别达1457.98万人次和150.12亿元，比增28.7%和29%。

　　农业增效农民增收有新进步。农业总产值、农村居民人均纯收入分别达256.91亿元和9036元，比增5.7%和15.5%。建成国家、省农业标准化示范区26个。新增省市农业（扶贫）龙头企业31家、农民专业合作社376个。新增省名牌产品9个，新认证无公害农产品、绿色食品、有机食品25个。梅州金柚、平远慈橙、嘉应茗茶等品牌知名度提高。

　　二、特色宜居城乡建设再上台阶

　　交通、水利等基础设施建设力度加大。梅大高速及其东延线扎实推进，汕湛高速五华段、济广高速平兴段动工建设，畲华、大潮高速前期工作有序推进；积极推动梅惠高铁开展前期工作；升级改造国、省道214公里，完成新农村公路建设650公里；梅县机场飞行区完成扩建并投入使用。韩江航道整治进展顺利。全年总投资28亿元的324宗重点民生水利工程顺利推进，梅州大堤南堤、西堤加固工程全面完成，五华、梅县成功竞得省级水利示范县，韩江（高陂）水利枢纽工程等重点水利项目前期工作稳步推进。投资65亿元的宝丽华荷树园电厂全面建成达产。投资15.5亿元的电网项目顺利实施。梅州（五华）抽水蓄能电站、粤电大埔电厂前期工作加快推进。

　　城乡规划和中心城区扩容有效推进。抓好《梅州市域城乡总体发展规划》、《梅州市城市总体规划》编制和各县县城规划修编工作。完成江北宜居城乡一期改造和江南新城控制性规划，大力推进芹洋半岛品牌创新运营区规划建设。梅县西阳镇划入梅江区，扩展了中心城区空间。梅县撤县设区工作有序推进。

　　城乡环境进一步优化。改造提升一批市政道路和城市广场，“拆围建绿”腾出城市绿地28万平方米，建成首段30公里绿道。大力淘汰水泥落后产能，推广LED 等高效节能照明产品，单位GDP能耗下降4.86%。成功申报国家级可再生能源建筑应用示范城市。饮用水源水质达标率和城市空气质量优良率均达100%。大力推进“一消灭三改造”工程，完成造林面积53.2万亩。重点林业生态工程建设受到省政府充分肯定。培训了一批农村建筑工匠，加强了农村建房管理。城乡环境卫生整治取得较好成效。

　　三、社会事业全面进步

　　积极推进文化惠民。自然村广播电视“村村通”工程全面完成。文化体制改革稳妥推进。大力推动优秀客家文化进校园、进社区。以我市道德模范彭彩金事迹为题材的励志电影《孝女彩金》在全国公映。新增省级非遗项目4个、省历史文化名镇2个和名村4个，9个村入选首批中国传统村落。五华狮雄山秦汉城址新一轮考古成果填补了南越国早期历史空白。兴宁获评 “中国围龙屋之乡”、“中国花灯之乡”。重视知识产权保护，市级机关事业单位在全省率先实现软件正版化。大力推进创建教育强市工作，新增教育强县2个、教育强镇11个，建成规范化学校60所。一批规模较大的职业（技工）学校办学水平提升。嘉应学院办学更具特色。学前教育、特殊教育和开放教育协调发展。深入开展“三打”专项行动，优化了投资发展环境，市场秩序明显好转；坚持打建并举，积极推进“两建”试点工作，探索建立具有梅州特色的社会信用体系和市场监管体系。创新社会管理，培育了一批“枢纽型”社会组织。“廉洁镇村”经验在全省推广，蕉岭县村务监督制度获广东治理创新奖。深入开展和谐劳动关系示范区和示范企业建设。严格落实安全生产“一岗双责”，安全生产形势持续稳定。平安梅州创建经验被省委省政府充分肯定。在全省公众安全感调查中，我市位居前列。抓基层、打基础、优服务，人口与计划生育工作有新进步。我市获“全国无偿献血先进市”称号。蕉岭籍运动员谢豪摘得伦敦残奥会金牌，梅县获省首届百县（区）足球赛总冠军。国防教育、人民防空和民兵预备役建设扎实推进。民族宗教、人事编制、妇女儿童、统计、物价、供销、气象、残联、打私、档案、方志等各项工作取得新成绩，外事侨务、港澳和对台工作更加务实灵活。

　　四、民生进一步改善

　　重点民生问题得到较好解决。财政支出进一步向民生倾斜，投入民生资金占公共财政预算支出的72.8%。2011年农村劳动力技能培训转移就业目标责任制考核获全省第一。全市共培训劳动力8.5万人，新增转移就业10万人，城镇登记失业率控制在2.4%以内，城镇居民人均可支配收入18699元，增长11.6%。全市镇（街）人社公共服务平台全面建成。县级公立医院和基层医疗卫生机构综合改革有效推进。“五大险种”综合参保193万人次，社保费征缴收入50亿元、比增50%。新型农村合作医疗和城镇居民医疗保险实现整合。32.3万农村群众饮水安全问题得到解决。保障性安居工程建设任务如期完成。

　　扶贫开发“双到”工作任务全面完成。通过省扶贫“双到”三年考核验收。贫困村集体收入全部达到4万元以上，4.4万户贫困户实现脱贫。完成农村低收入住房困难户住房改造12828户，20个省级 “幸福安居工程”示范村建设扎实推进。

　　十件民生实事全面实施。全市60周岁以上城乡居民全部享受养老保险；梅州城区小学生、60岁以上老人和残疾人免费乘坐梅城公交车；市实验小学、市田家炳医院心脑血管诊疗中心、市残疾人康复服务中心、黄塘河综合整治工程和城区污水处理厂二期工程动工建设；205国道梅城江北段提升改造工程顺利推进；梅州城区、梅揭高速公路等主要干道300公里生态景观林带建设任务全面完成；客商会馆和客都展馆新址初步选定。

　　五、政府自身建设加强

　　“六五”普法深入开展，法治县（市、区）创建工作成效明显。积极推进政府行政复议委员会试点工作。大力推进“审管分离”部门内部行政审批职能相对集中改革，市级行政审批时限和事项实现“双减半”。企业登记审批制度改革试点工作有序推进。政府购买服务范围扩大。重点领域、重点项目实行同步监督和重点审计，全国性社保基金审计等项目高质量完成。认真落实党风廉政建设责任制。市政府在省党风廉政建设责任制考核中被评为“好”的等次。自觉接受人大及其常委会的监督和政协民主监督，主动向人大及其常委会报告工作、向政协通报情况，74件代表建议、180件政协提案全部办复。

　　各位代表！上述成绩来之不易，这是在省委、省政府和市委领导下，全市人民克难攻坚、奋力拼搏的结果。在此，我谨代表市政府，向全市人民、中央和省属驻梅单位、驻梅部队，向所有关心和支持梅州发展的海内外同胞们、朋友们，表示衷心的感谢并致以崇高的敬意！

　　在看到成绩的同时，我们也清醒地认识到，我市经济社会发展还存在不少困难和问题：一是经济实力弱，产业层次低，加快发展、创新发展和提升发展的任务艰巨。二是交通不发达，对接珠三角、汕潮揭和厦漳泉的快速交通网络未建成，交通“瓶颈”未突破。三是文化事业发展滞后，客家文化保护与传承需加强。四是城乡基本公共服务均等化水平低，民生工作与群众期望有差距。五是公务员队伍欠缺改革创新、攻坚克难的锐气、勇气和韧劲，等等。对这些问题，我们将高度重视，采取措施，认真解决。

　　2013年工作安排

　　2013年，是全面贯彻党的十八大精神的开局之年。去年底，习近平总书记视察广东并发表重要讲话，对广东提出“三个定位、两个率先”的总要求，为全省经济社会发展注入了强大动力。省委、省政府更加重视区域协调发展，并将专题研究部署支持山区加快发展。全市广大干部群众必须牢固树立加快山区绿色崛起的机遇意识和责任意识，切实增强紧迫感和危机感，抓住机遇，扎实工作，埋头苦干，加快发展。

　　今年政府工作的总体要求：深入贯彻党的十八大和中央、省委、市委系列会议，以及习近平总书记在粤考察，胡春华书记来梅调研重要讲话精神，大力实施“一园两特带动一精”发展战略，勇于改革创新，增强发展动力；狠抓交通建设，突破发展瓶颈；注重实业强市，实施产业振兴；强化投资拉动，发展市域经济；统筹城乡发展，建设生态文明；加强文化建设，增创后发优势；全力加快绿色经济崛起，为尽快赶上全国平均水平打牢基础。

　　今年全市经济社会发展的主要预期目标：生产总值增长12%；公共财政预算收入增长15%；固定资产投资增长27%；单位生产总值能耗下降3.89%；城镇居民人均可支配收入增长10%；农村居民人均纯收入增长10%；人口自然增长率控制在9.3‰以内。

　　根据上述目标任务，我们重点抓好七方面工作。

　　一、全力推进基础设施建设

　　抓重大项目、重大平台建设。切实加大投资力度，推进169项重点项目建设。抓好59个现代产业项目、29个社会事业项目和一批电网项目的落实。致力推进“三区三城”建设。抓好低丘缓坡荒滩等未利用地开发利用试点工作，加大“三旧”改造和闲置土地处置力度，盘活存量建设用地。及时协调解决好项目用地、资金、征地拆迁等问题，确保重大项目顺利推进。

　　开展交通建设大会战。全力推进梅大高速及其东延线、汕湛高速五华段、济广高速平兴段建设。力促畲华高速和梅漳高速尽快立项建设。抓紧做好大埔至潮州港、大埔经丰顺至揭西、梅州经潮州揭阳至汕头港高速公路和梅惠高铁、梅州至厦深高铁连接线，鹰梅、浦梅铁路及汕潮揭城际轻轨丰顺延长线的前期工作。抓好国、省、县道和县际联络线及旅游干线建设。扎实开展梅县机场迁址扩建的前期工作。继续实施韩江航道整治工程。

　　抓好重点水利工程建设。大力推进268宗重点民生水利建设，力促韩江（高陂）水利枢纽工程尽快动工，加快梅南水利枢纽工程、梅县松口古镇防洪工程、兴宁罗浮调水工程、平远凤池水库等重点水利项目前期工作。组织好兴宁、五华、梅县等省级水利示范县建设。改造江北老城区自来水管网，完成城区污水处理厂二期工程。

　　二、大力推动新型工业化

　　坚持发展实业和实体经济。抓紧制订发展总部经济政策。实施工业振兴三年行动计划，大力推动传统优势产业转型升级。推动烟草产业做优做强，力争两年内实现产值超百亿税收超五十亿；鼓励BPW 车轴、广汽强华、荻赛尔等重点企业增资扩产、技改挖潜，做大汽车零部件产业；发展中高端电路板、电声整机等电子信息和电声产业；致力名牌带动、技改升级，发展绿色建材产业；推进梅州（五华）抽水蓄能电站、粤电大埔电厂等项目建设，发展绿色能源产业；大力推进资源整合和精深加工，提高稀土、钼矿等开发效益；以大埔高陂陶瓷生产基地为龙头，做精做强特色陶瓷产业；以厚工坊、长乐烧、一线天和客家娘酒等品牌为纽带，推动酿酒业整合升级。力促宝丽华、梅雁、塔牌、嘉应制药、超华科技等骨干企业做大做强。继续扶持有条件的企业上市或进入区域股权交易中心融资发展。实施“客商”培养计划。设立市政府质量奖，推动质量强市深入开展。

　　提升高新技术产业园区建设水平。进一步优化园区规划，全面推进二期主干道路、供电、给排水管网和生活配套区建设，不断增强园区的承载能力和集聚能力。大力推动国威电子、伊利冷饮、科伦药业、三一重工等在建项目建设。创新园区管理体制和办园机制，力争引进战略合作和专业投资经营者。搭建一批公共创新服务平台，提升园区产业竞争力。

　　狠抓招商引资。深入实施乡贤回乡投资兴业工程，打好招商引资大会战。突出产业链招商和以商引商，着力引进一批投入强度大、综合效益好的先进制造业和先进服务业项目，突出项目管理，力促金发科技、毅昌科技、颐高电商、海吉星物流、华科国际、中凯文化等具有行业竞争力的企业落户。发挥著名侨乡和“海峡两岸交流基地”的优势和效应，用好海内外客家人的人脉资源，加大对外和对台港澳招商引资力度。加强与境外企业以及中央、省属企业和知名民企的合作交流。充分利用各类行业协会、商会和客属团体招商，办好第二届客家文化艺术节暨世界客商大会，拓宽招商引资渠道。

　　推动信息化、工业化、城镇化和农业现代化协同发展。实施梅州信息产业发展三年行动计划。加强信息基础设施建设，推动信息网络技术在经济、政治、文化、生态建设和社会管理各领域的广泛应用。加快联通粤东通信枢纽中心、梅州移动生产调度中心建设。推动信息化与工业化深度融合，工业化与城镇化良性互动，城镇化与农业现代化相互协调，促进城乡产业协调发展。推进信息技术与城市建设管理的深度融合，打造“智慧客都、数字梅州”。

　　三、着力发展幸福导向型产业

　　推进广东梅州文化旅游特色区建设。抓住国家实施国民旅游休闲纲要的机遇，大力推广健康、文明、环保的旅游休闲理念，促进旅游休闲的规模扩大和品质提升。抓紧编制特色区发展总体规划，制定幸福导向型产业发展指导目录。抓好雁洋生态文化旅游产业园、客家文化影视基地、客天下旅游产业园和叶帅故园、富力足球学校等一批重点项目建设。积极培育发展旅游度假、保健疗养、健康管理、婚庆服务、低碳环保、文化创意等幸福导向型产业。加大对旅游的整体策划和营销推介，拓展中高端市场。大力开发文化旅游商品，提升旅游产业增加值，延长旅游产业价值链。

　　积极发展电子商务和现代物流业。实施电子商务和现代物流三年发展计划，落实产业扶持政策。大力发展新型流通业态和营销模式，引进有行业影响力的企业建设物流产业园区，建立结构合理的物流配送体系。策划引资建设电子商务产业园，争取引进阿里巴巴等电商龙头企业。多渠道扩大消费，鼓励中小民营企业参与投资电子商务，搭建网上商城，组织企业参加 “广货全国行”、“广货网上行”等活动，积极拓展市场；把握休闲度假和健康养老产业的发展趋势，引导房地产业创新发展、健康发展。依托嘉应学院和职业（技工）学校，培养电子商务专业人才和管理人才。积极推动电子商务学院规划建设，为电子商务和物联网发展提供人才支撑。

　　发展文化创意产业。出台鼓励扶持文化产业发展政策，探索建立文化产业投融资、经纪推介和交易服务等平台，发展文化博览、创意设计、艺术创作表演、工艺美术和动漫游戏等产业，推动客家文化资源市场化开发和专业化运作。推进麓湖山文化产业园、东山教育文化基地和千佛塔等宗教文化区建设。力争引进中凯文化科技博览园等优质文化产业项目。举办全市文化旅游工艺品设计大赛和客家文化创意博览会。引进中高端商业演出，培育文化消费市场。加快广东汉剧院、市山歌剧团等文艺院团的转型，打造特色文化精品，提高市场竞争能力。策划拍摄以叶剑英、张弼士和李惠堂等客家名人为题材的影视作品，提升梅州历史人文的影响力。

　　四、提升“三农”工作水平

　　着力发展精致高效农业。推进梅县台湾农民创业园和市农业科技创新中心建设。推动省市共建绿色生态水产创新示范园。建设雁洋金柚公园等一批休闲观光农业基地。培育一批有较强带动能力的农业龙头企业和农民专业合作社。鼓励民营资本进入农业开发领域，打好耕山致富大会战。支持发展家庭农场、家庭林场、民营庄园等新型农业经营主体。建设一至两个农产品物流配送中心或农产品物流园。强化农产品地理标志和商标保护，进一步打响梅州金柚、平远慈橙、嘉应茗茶等优质农产品品牌。推进农业标准化建设，提升农产品安全水平。加强农业科技工作，积极开展与高等院校和科研院所的合作，培养农业科研队伍，力争在良种良法研究推广和农产品精深加工方面有较大突破，促进农民增收，农业增效。

　　改善农村生产生活条件。扎实推进基本农田建设，开展中小河流治理、小流域综合整治、中小型灌区改造和病险水库除险加固，提高山洪、地质灾害预警预报水平。全面落实农业补贴优惠政策。积极稳妥推进宅基地使用权和土地承包经营权抵押贷款试点，推动农村金融改革，更好地服务“三农”工作。加强农村集体“三资”管理。加快户籍制度改革。高度关注农村留守儿童、孤残儿童、单亲特困母亲和老退伍兵等群体，各级政府要注入资源，带动社会力量，切实保障这些特殊困难群体的基本权益。

　　推进新一轮扶贫开发工作。认真实施 《梅州市农村扶贫开发实施意见》，突出抓好抓实产业扶贫和技能扶贫。规划建设一批有地方特色的产业扶贫示范点，以点带面，引导群众脱贫致富。实施新一轮农村低收入住房困难户危房改造，完成1.16万户改造任务。大力推进“两不具备”村庄搬迁工作。支持原中央苏区县获得更多的政策、资金和项目扶持。

　　五、建设美丽城乡

　　大力推进新型城镇化。按“城是宜居区，乡是生态园”的规划理念，继续推进 《梅州市域城乡总体发展规划》编制。按照建设粤闽赣边区域性中心城市的定位和功能要求，修编《梅州市城市总体规划》，推进中心城区扩容，致力抓好江南新城、梅县新城、畲江高新技术产业园和雁洋文化旅游综合服务区等规划建设。按“精致慢城”的内在要求，强化城市的精细管理，提升城市综合素质，让旅客感受到我们的城市有亲和力和人情味，让市民真切地感受到自己生活的城市有安全感和幸福感。加快“大百汇品牌发展创新园”规划建设，打造区域总部经济基地。抓好丰顺新区、兴宁新城、各县城和中心镇建设，加快城镇化进程。

　　积极推进幸福村居建设。逐步完善城乡规划体系建设，提高镇村规划覆盖率。加大农村建筑工匠培训力度，引导农民依规建房。组织好城乡环境整治大会战，全面开展“清洁家园”和“万村绿”创建活动，清理垃圾、疏浚河道、清洁城乡。推进市、县和中心镇污水处理设施建设，提高城乡环境卫生管理质量和水平。抓好一批美丽乡村示范点，建设一批富有客家特色的名镇名村。

　　优化生态环境。建立完善生态保护制度，实施以封管为重点的“森林安保”行动。全面推进“一消灭三改造”，完成造林50.85万亩。积极研究和探索建立森林碳汇交易制度。大力倡导绿色消费，全面促进资源节约，加强节能减排，提高资源利用的效益和效率。实行最严格的耕地保护制度和水资源保护制度。动员全市力量，共同创建国家环境保护模范城市。

　　六、努力推进基本公共服务覆盖城乡

　　促进更高质量的就业。以市场需求为导向，整合职业培训资源，加快完善公共就业和人才服务体系，推动校园与产业园对接，大规模开展职业技能培训。依托各级公共就业和人才服务机构，为群众提供更好更有效的就业服务。

　　逐步提升社会保障水平。进一步推进覆盖城乡居民的养老保险、医疗保险制度建设，深化医药卫生体制和食品药品监管机制改革，鼓励支持民营医院发展，推进丰顺和蕉岭县级公立医院综合改革试点工作。逐步提高城乡低保标准，完善社会救助体系，保障城乡困难群众、低收入者和特殊困难人员的基本生活。扶持发展民办养老机构。建设保障性住房1000套，年内竣工720套。

　　推动教育均衡发展。围绕创建“广东省教育强市”合理配置教育资源，因地制宜抓好学校和幼儿园建设。通过以点带面全面提高普及高中阶段教育水平。整合职业（技工）学校，引导职业技术教育规模化、规范化、特色化发展。创造条件办好学前教育、特殊教育和民办教育。以嘉应学院和东山中学百年校庆为契机，动员社会资源，推动高等教育和高级中学办学水平提高。坚持立德树人理念，落实课程改革，推进素质教育，促进学生全面发展、健康成长。发挥“嘉应名师”评选表彰活动效应，加强师资队伍建设。

　　发展文化体育侨务等社会事业。大力弘扬新时期广东精神和优秀客家传统文化，加强社会主义核心价值体系建设，不断提升城乡居民思想道德素质。抓好抓实文物保护等发展文化事业的基础性工作，认定一批市、县文物保护单位，启动对可移动文物的普查工作。抓紧推进围龙屋申报世界文化遗产工作，实施客家文化生态保护实验区规划，加强客家山歌、广东汉剧等“非遗”的保护和传承。利用围龙屋、老祖屋建设农村综合文化社区。广泛开展全民健身运动，大力实施振兴足球“十个一”工程，打响梅州“足球之乡”品牌。重视做好新时期外事侨务和港澳台工作，凝心聚力推动梅州加快发展、科学发展。稳定低生育水平，促进人口均衡发展。继续扎实推进平安梅州建设。全面落实安全生产“一岗双责”制度，有效防范和遏制安全生产事故。重视解决农村学生的上学交通安全问题。统筹推进国防建设、气象、防灾减灾、民族宗教和政府法制、人事编制以及统计、方志、档案等各项工作再上台阶。

　　七、全面加强政府建设

　　加强法治政府建设。坚持科学决策、民主决策、依法决策，出台重大决策听证、论证和咨询制度，确保政府的重大决策都能广纳民意，重要行政决定都能普惠民生。落实好向人大及其常委会报告工作和向政协通报情况制度。办好人大建议和政协提案。认真听取和采纳各民主党派和社会各界意见，加强政务、村务公开，主动接受社会监督。

　　加快转变政府职能。按建设“大社会、好社会，小政府、强政府”的方向，深化行政体制改革。扎实推进第六轮行政审批制度改革，市级第一季度、县级上半年基本完成审批事项的清理工作。年内完成县级大部制改革。以企业登记审批制度改革为突破口，全面推进市场监管体系和社会信用体系建设，构建法治化国际化营商环境。制定扶持社会组织发展计划，重点培育行业管理、公益慈善、社区工作、产业协会等枢纽型社会组织。编制政府向社会组织转移职能目录和购买服务目录，大力发展社会服务业。推动志愿服务事业发展，在全社会培育和弘扬志愿服务精神。

　　强化政府作风建设。认真落实中央改进工作作风、密切联系群众八项规定。厉行勤俭节约，反对铺张浪费，严格控制“三公经费”支出。扎实做好服务基层、服务企业、服务群众工作。严格落实党风廉政建设责任制，深化惩防腐败体系建设，加强对重点项目、重点资金和重大工程的审计检查。加强公务员教育培训，提高培训绩效。扎实开展“为民、务实、清廉”为主题的群众路线教育活动，着力治理庸、懒、散、奢，切实提高政府机关依法办事效能和整体服务水平。

　　各位代表，梅州事业的发展依靠人民，发展的成果应由人民共享。今年我们将全市动员、上下联动，集中力量办好十件惠民实事：

　　一是就业惠民。确保有培训意愿、有转移就业意愿的新成长劳动力100%参加培训和转移就业；全年培训劳动力8万人次，转移就业10万人次；新增城镇就业3万人，城镇失业登记率控制在3.5%以内。

　　二是教育惠民。力争成为全省山区首个教育强市；继续实施“嘉应名师”工程，招聘一批优秀博士、硕士研究生到我市任教；推进学生饮用奶计划，通过财政补贴和社会资助，让特殊困难学生免费喝上学生奶。

　　三是文化惠民。建设嘉应歌剧院；建设县级图书馆1个、博物馆1个，镇村文化站（室）、电子阅览室330个；完成未通有线网络的农村地区广播电视“户户通”工程。

　　四是健康惠民。实施城乡居民大病保险制度，切实减轻城乡居民医疗费用负担；建立1家以上平价医院和20家平价药店；实施免费孕前优生健康检查。五是食品安全。评选1000家流动食品安全示范点；整合检验检测资源，成立市食品安全检验检测中心；成立1家“三星级”肉联厂和新建1家新型现代化屠宰场，使广大群众吃上“放心肉”。

　　六是交通惠民。建设梅州市中心枢纽汽车站；完成新农村公路建设400公里；梅城公交车免费乘坐范围扩大至城区初中学生。

　　七是环境惠民。以绿化高速公路、国省道等主干道沿线和宜林荒山、江河两岸为重点，完成森林碳汇重点生态工程建设50万亩；动工建设环保能源（垃圾焚烧）发电项目；建设改造兴宁、平远、蕉岭、大埔、丰顺、五华生活垃圾无害化处理场；深入开展城乡环境卫生整治，净化美化亮化城乡环境。

　　八是水利惠民。完成各县（市、区）17宗山区小型灌区改造工程建设项目，改善农田灌溉面积42830亩，实现旱能灌、涝能排，惠及18个镇48条村12.6万人；实施7宗投资1.2亿元的中央和省小型农田水利重点县建设项目；解决约8万农村人口饮水安全问题。

　　九是养老助困。全市月人均低保补差水平达到城镇248元、农村116元；农村五保供养标准不低于当地上年农村居民人均纯收入的60%；为200户贫困家庭残疾人实施居家无障碍环境改造；为200户贫困家庭残疾人提供康复服务；建设2-3个城市居家养老服务示范中心，改造一批乡镇敬老院，新增养老床位2800张。

　　十是政务惠民。完成县级行政服务中心建设，建成市县两级网上办事大厅，实现网上政务“一站式办理、一条龙服务”，提高办事效率，方便群众办事。

　　各位代表，要让美好的蓝图变成现实，需要有坚毅执着、一往无前的坚定信念，需要踏石留印、抓铁有痕的务实作风，更需要全市干部群众脚踏实地、埋头苦干的奉献精神。让我们在中共梅州市委的领导下，改革创新，锐意进取，抓住机遇，真抓实干，全面完成今年的工作任务，力争交上一份让全市人民满意的答卷！
